# T-CD4+ cell count at the date of the HIV diagnosis

**DOI:** 10.1186/1758-2652-13-S4-P175

**Published:** 2010-11-08

**Authors:** AR Domingues da Silva, J Mendez, R Sarmento e Castro

**Affiliations:** 1 Hospital de Joaquim Urbano, Infectious Diseases, Porto, Portugal

## Background

Some studies state that HIV patients' immunological stage at the date of diagnosis has not been significantly altered in the last few years despite a better access to health care, and the innumerable campaigns for the detection of infection due to HIV. Others come to the conclusion that the mean T CD4+ cells count has been reducing since 1985 but no explanation for this fact has yet been found.

## Purpose of the study

Evaluation of the influence of epidemiological parameters and year of diagnosis on the immunological stage of the HIV infected patients at the time of diagnosis.

## Methods

Retrospective analysis of a random survey of the clinical files of HIV positive patients followed at Hospital de Joaquim Urbano, from 1995 to 2008.

## Summary of results

730 files have been analysed. 80.1% of these patients were male and 19.9% female. The average age at HIV diagnosis was 36 years (minimum 14, maximum), 1.9% where under 20 years old and 4.2% over 60 years old. 59.3% were intravenous drug users, 26.6% heterosexuals, 6.2% homosexuals and in 7.3% of the patients the risk factor for acquisition of infection was unknown. Of the total, 38.2% were HIV mono-infected, 48.2% where HIV/HCV; 9.2% HIV/HCV/HBV and 4.4% HIV/HBV co-infected. The global mean T CD4+ cell count at the time of diagnosis was 293/mm^3^. There were no significant statistical differences (p>0.05) between gender: male 289.5/mm^3^ and female 305.8/mm^3^, nor age at the time of diagnosis. The mean TCD4+ cell count was higher in the homosexual patients (391.9/mm^3^) when compared with heterosexuals (273.9/mm^3^) and iv drug users (300.7/mm^3^) (p<0,05). The mean TCD4+ cell count at diagnosis was: 306/mm^3^ in HIV mono-infected patients, 288.2/mm^3^ in HIV/HBV, 285.4/mm^3^ in HIV/HCV and 278.2/mm^3^ in HIV/HCV/HBV co-infected patients, although these differences are not statistically significant among them. At diagnosis a more severe immunosuppression (CD4<200/mm^3^) was registered in 48.7% of the male population, 53.8% of patients over 60 years old, 54.1% of the heterosexuals, and 62% of VIH/VHB co-infected. When comparing the mean T CD4+ cell count between 1995-1997 (432/mm^3^), and 2005-2008 (246/mm^3^) significant statistical differences were found (p<0.05).

## Conclusions

The homosexual group of patients presented a better immunological stage at the date of diagnosis. In the remaining groups there were no significant statistical differences in the immunological stage when the HIV infection.

